# Mixing Enthalpies of Liquid Ag–Mg–Pb Alloys: Experiment vs. Thermodynamic Modeling

**DOI:** 10.3390/ma15207360

**Published:** 2022-10-20

**Authors:** Adam Dębski, Władysław Gąsior, Wojciech Gierlotka, Marek Polański

**Affiliations:** 1 Institute of Metallurgy and Materials Science, Polish Academy of Sciences, 25 Reymonta Street, 30-059 Cracov, Poland; 2 Department of Materials Science and Engineering, National Dong Hwa University, Hualien 97401, Taiwan; 3 Department of Functional Materials and Hydrogen Technology, Military University of Technology, 2 Kaliskiego St., 00-908 Warsaw, Poland

**Keywords:** Ag–Mg–Pb, enthalpy, thermodynamic properties, calorimetry, thermodynamic modeling

## Abstract

A drop calorimetric method was used to measure liquid Ag–Mg–Pb alloys. The partial and integral mixing enthalpies of the investigated alloys were determined at a temperature of 1116 K. The experiments were performed for four separate series starting from binary alloys with a constant *x*_Mg_/*x*_Pb_ ratio of 1/3, 1, 3 ((Mg_0.25_Pb_0.75_)_1−x_Ag_x_, (Mg_0.50_Pb_0.50_)_1−x_Ag_x_, (Mg_0.75_Pb_0.25_)_1−x_Ag_x_) and *x*_Ag_/*x*_Mg_ ratio of 1/3 (Ag_0.25_Mg_0.75_)_1−x_Pb_x_. Next, the ternary interaction parameters were determined using the Muggianu model, the thermodynamic properties of binary systems in the form of the Redlich-Kister equations and the values of the mixing enthalpy changes, which were determined in this study. The partial mixing enthalpies of Ag, Mg, and Pb were calculated based on the binary and elaborated ternary interaction parameters for the same intersections in which the measurements were conducted. It was found that the ternary Ag-Mg-Pb liquid solutions are characterized by negative deviations from the ideal solutions, with a maximal value slightly lower than –13 kJ/mol for alloys with the ratio (Mg_0.75_Pb_0.25_) and *x*_Ag_ = 0.4166.

## 1. Introduction

Knowledge of phase equilibria, existing intermetallic compounds, melting temperatures, and invariant reactions is essential for people who design new materials. The above information can be read from the phase diagram or calculated if a suitable thermodynamic model is available. Therefore, careful examination of phase equilibrium systems, starting from binary systems, is extremely important from the point of view of the development of science itself. In recent years, there has been an intensive development of magnesium-based alloys, which, thanks to their properties such as their low density, high specific strength, and good castability [1], have been widely used in many areas of industry. For example, the binary Mg–Pb alloy was proposed as a cooling medium for nuclear fast reactors [2] or as a novel material for solar cells [3]. Moreover, in recent years, due to their ability to interact with hydrogen, these alloys have been studied as materials that safely store hydrogen [4,5]. The addition of silver has a positive effect on the properties of Mg-based alloys [6] since it was found to possess catalytic properties for the dissociation of molecular hydrogen, as well as potential to protect metals from surface oxidation [7]. For these reasons, and also cognitive motivation, in two of our previous works, we investigated the ability of Mg–Ag alloys produced by mechanical milling [8] and classical melting and casting [9] to absorb hydrogen. The addition of Pb to this system is not beneficiary from a density point of view since it rather lowers the gravimetric hydrogen capacity of such alloys. However, lead possesses a significantly higher atomic radius than both Mg and Ag. This means that it may cause a significant lattice expansion when added as an alloying element. Expansion of the lattice may lower the equilibrium pressure of the hydrogen absorption and by that allow easier hydrogenation of the alloy.

A literature review concerning thermodynamic studies of Ag–Mg and Mg–Pb systems was presented in our previous papers [10,11,12]; therefore, only information about thermodynamic studies of the Ag–Pb system will be provided in this paper.

The Ag–Pb phase diagram is a simple eutectic system and has been widely investigated [13,14,15,16,17,18,19]. A detailed review can be found in [16]. The liquidus line has been determined by several techniques, such as thermal analysis, metallography, and diffusion studies [20,21,22,23,24,25]. The mutual solid solubility of Ag and Pb was reported previously [26,27,28,29].

The enthalpy of mixing of Ag–Pb liquid alloys has been measured many times by the calorimetric method and conducted at different temperatures. Kawakami performed measurements at 1323 K [30], Kleppa at 723 K [24], Von Samson-Himmelstjerna at 773 K [31], Ehrlich at 1248 K [32], Kozuka et al. at 1273 K [33], Castanet et al. [34] at 1280 K, Itagaki and Yazawa [35] at 1243 K, and Hultgren and Sommelet at 1250 K [36]. The obtained mixing enthalpy had a positive deviation from ideal solutions. The values obtained by Castanet et al. [34] and Kozuka et al. [33], which were measured at approximately the same temperature, are consistent but differ significantly from the data presented by Itagaki and Yazawa [35]. Hultgren and Sommelet’s data [36] are less endothermic than those measured by Castanet et al. [34]. Kawakami’s data [30] measured at 1323 K differ significantly from the data presented by Von Samson-Himmelstjerna [31] at 773 K. This may suggest that the mixing enthalpy of Ag-Pb alloys is temperature dependent.

Thermodynamic activities in liquid alloys have been determined by many researchers using the emf method [37,38,39,40,41] and vapor pressure measurements [42,43,44].

Since calorimetric measurements of the mixing enthalpy of ternary Ag–Mg–Pb solutions have not yet been carried out, in this study, high-temperature drop calorimetric studies were performed to determine the partial and integral mixing enthalpies of the liquid mentioned above for the ternary alloys. This work initiates research on the thermodynamic properties of Ag–Mg–Pb solutions, and it is the first step of the investigation before modeling the phase equilibria in this ternary system.

## 2. Materials and Methods

All calorimetric measurements were performed in a high purity argon atmosphere using magnesium oxide (MgO) crucibles. All experimental series were performed with the use of the Setaram MHTC 96 Line Evo calorimeter. The samples were prepared from the high purity metals presented in Table 1, whose shape was similar to a sphere about 3 mm in diameter or a cylinder 3 mm in diameter and 3 to 20 mm high. Before the beginning of each measurement series, the calorimeter was evacuated several times with the use of a vacuum pump and flushed with high purity argon. Before being dropped into the reaction crucible, the samples were mechanically cleaned with a file to remove the possible surface impurities. After stabilizing the temperature and the baseline, the calibration constant was determined. Lead samples were used to determine the calibration constant in experimental series 1–3, while in series 4, silver samples were applied. The measured thermal effect was studied five times during the calibration process. In each measurement series, the following parts can be distinguished: the calibration process, measurement of the mixing enthalpy for a starting binary alloy, and measurement of the mixing enthalpy for ternary alloys. Each of the above-mentioned parts of the measurement may be presented as follows:

(a) The calibration process:

for series 1–3: *z*Pb_(s, TD)_ → *z*Pb_(l, TM)_ or for series 4 *x*Ag_(s, TD)_ → *x*Ag_(s, TM)_;

(b) Measurement of the mixing enthalpy of binary alloys:

Series 1–3: *z*Pb_(l, TM)_ + *y*Mg_(s, TD)_ → Mg_y_Pb_z(l, TM)_ or Series 4: *x*Ag_(s, TM)_ + *y*Mg_(s, TD)_ → Ag_x_Mg_y(l, TM)_

(c) Measurement of the mixing enthalpy of ternary alloys:

Series 1–3: Mg_y_Pb_z(l, TM)_ + *x*Ag_(s, TD)_ → Ag_x_Mg_y_Pb_z_^i^_(l, TM)_;

Series 4: Ag_x_Mg_y(l, TM)_ + *z*Pb_(s, TD)_ → Ag_x_Mg_y_Pb_z_^i^_(l, TM)_; where *T*_D_ and *T*_M_ are the drop and measurement temperatures, respectively; “s”, and “l” denote the solid (crystalline) and liquid states, respectively; x, y, z are the number of moles of Ag, Mg, or Pb; Mg_y_Pb_z(l, TM)_ or Ag_x_Mg_y(l, TM)_ symbolizes the formation of a starting binary Mg–Pb or Ag–Mg alloy and includes the increments of enthalpy of the pure magnesium and the melting enthalpy at the measurement temperature; and Ag_x_Mg_y_Pb_z_^i^_(l, TM)_ symbolizes the formation of the *i*-th ternary alloy (*i* = 1, 2, 3, …) and includes the increments of enthalpy of the pure dropped metal (Ag or Pb) and the melting enthalpy of the dropped metal at the measurement temperature.

The equations listed below were used to calculate the integral enthalpies of mixing (∆_mix_*H*) of the Ag–Mg–Pb liquid alloys:

(1) ΔHmix=∑ HDISS−XnAg+nMg+nPb

(2) $$H_{\mathrm{DISS}-X}=\left(\Delta H_{\mathrm{Signal}}\cdot K\right)-\left(\Delta H_{X}^{T_{D}\to T_{M}}\cdot n_{X}\right),$$

(3) $$K=\frac{\Delta H_{X}^{T_{D}\to T_{M}}\cdot n_{X}}{\Delta H_{\mathrm{Calibration}}}$$

where $\Delta H_{\mathrm{Signal}}$ is a voltage signal given in µV/s that is caused by the heat increment originating from each dropped metal (Ag or Pb); *K* is the calibration constant; $\Delta H_{X}^{T_{D}\to T_{M}}$ is the molar enthalpy difference of X element (X = silver and lead) between room temperature (*T*_D_ = 298 K) and the temperature of measurement (*T*_M_), which was calculated using [45]; $n_{x}$ ($n_{\mathrm{Ag}},\;n_{\mathrm{Mg}},\;n_{\mathrm{Pb}}$) is the number of moles of silver, magnesium, and lead, respectively; $H_{\mathrm{DISS}-X}$ is the enthalpy of dissolution of pure silver and lead; and $\Delta H_{\mathrm{Calibration}}$ is the voltage signal given in µV/s that is caused by the heat increment originating from the dropped lead sample, which was used for calibration.

## 3. Results and Discussion

**Figure 1:**
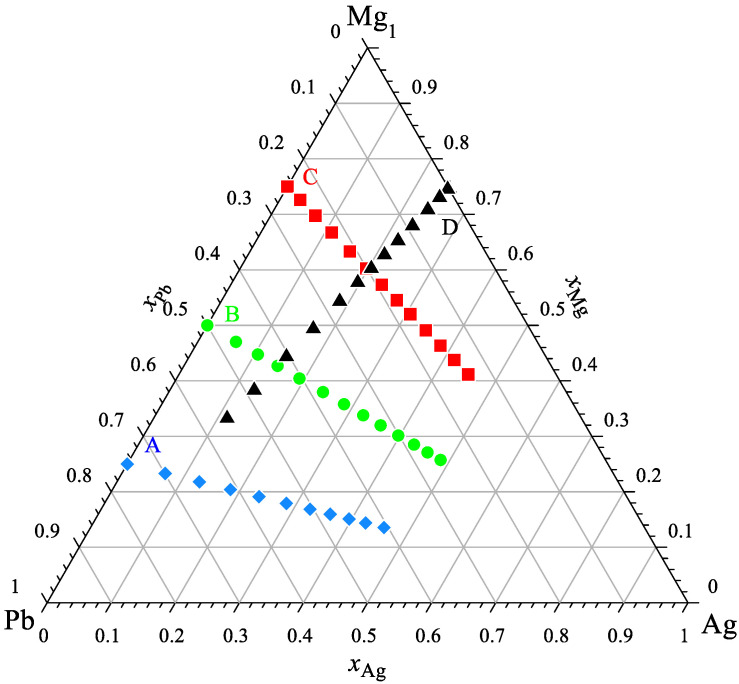
The studied compositions of alloys from the Ag-Mg-Pb system.

The calorimetric study of the Ag-Mg-Pb system was conducted at four separate experimental series for a constant ratio of *x*_Mg_/*x*_Pb_ equal to 1/3, 1, 3 ((Mg_0.25_Pb_0.75_)_1−x_Ag_x_, (Mg_0.50_Pb_0.50_)_1−x_Ag_x_, and (Mg_0.75_Pb_0.25_)_1−x_Ag_x_) and *x*_Ag_/*x*_Mg_ equal to 1/3 ((Ag_0.25_Mg_0.75_)_1−x_Pb_x_) to verify the obtained results. The compositions of all investigated alloys in these studies are shown in Figure 1.

The calorimetric studies were performed at 1116 K for four series with constant ratios of *x*_Mg_/*x*_Pb_ and *x*_Ag_/*x*_Mg,_ as mentioned above. The obtained experimental values of the heat effects, the integral molar mixing enthalpy of liquid Ag–Mg–Pb alloys, the mole fraction of elements, the drop enthalpies and other information measured in these studies are listed in Table 2 and Table 3.

The calorimetric data of the mixing enthalpy change of Ag–Mg–Pb liquid alloys, presented in Table 2 and Table 3, were used to elaborate the thermodynamic properties (∆_mix_*H*) of liquid Ag–Mg–Pb solutions by the Muggianu model [46], with the additional mathematical expression describing the ternary interactions. In such a case, this model can be expressed as follows:

(4) ΔmixH=∑i∑j>i(xi·xj·∑kLki,jLiquid(xi−xj)k)+xixjxk·(L0123Liquid+L1123Liquid·xi+L2123Liquid·xj+L3123Liquid·xk) 

The parameters in Equation (4) are marked as follows: $\Delta_{\mathrm{mix}}H$ is the mixing enthalpy change of the liquid Ag–Mg–Pb alloys; $x_{i},\;x_{j},\;x_{k}$ are the Ag, Mg, and Pb mole fractions, respectively; Lki,jLiquid are the binary interaction parameters in the Redlich–Kister polynomial [47] for the Ag–Mg, Ag–Pb, and Mg–Pb binary systems; and Lk123Liquid (*k* = 0, 1, 2, 3) are the ternary interaction parameters. More information on this topic can be found in [48].

Using the obtained calorimetric data of the mixing enthalpy change for the Ag–Mg–Pb liquid solutions, the Lk123Liquid parameters were calculated by the least square method by utilizing the optimization own computer program (TerGexHm). The calculated standard deviation is equal to 582 J/mol, and the values of all parameters in Equation (4) are shown in Table 4.

**Figure 2:**
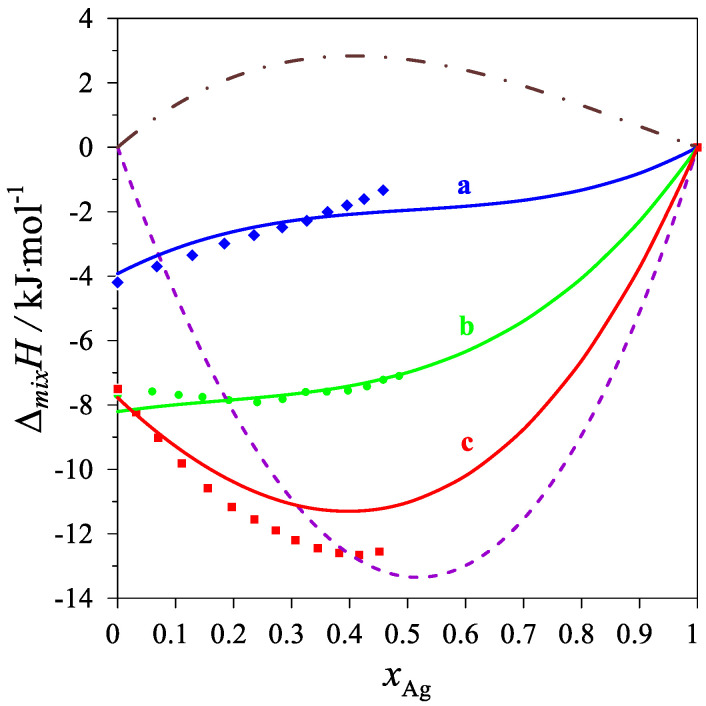
The integral mixing enthalpy of liquid Ag-Mg-Pb alloys at 1116 K, for the following intersections: (a)—Series A (Mg_0.25_Pb_0.75_)_1−x_Ag_x_—blue line: data calculated with Equation (4), blue diamonds—experimental data; (b)—Series B (Mg_0.50_Pb_0.50_)_1−x_Ag_x_—green line: data calculated with Equation (4), green circles—experimental data; (c)—Series C (Mg_0.75_Pb_0.25_)_1−x_Ag_x_—red line: data calculated with Equation (4), red rectangles—experimental data. The purple dash line is the integral molar mixing enthalpy of Ag–Mg [10] liquid alloys, and the brown dash-dot line is the integral molar mixing enthalpy of Ag–Pb [17] liquid alloys.

**Figure 3:**
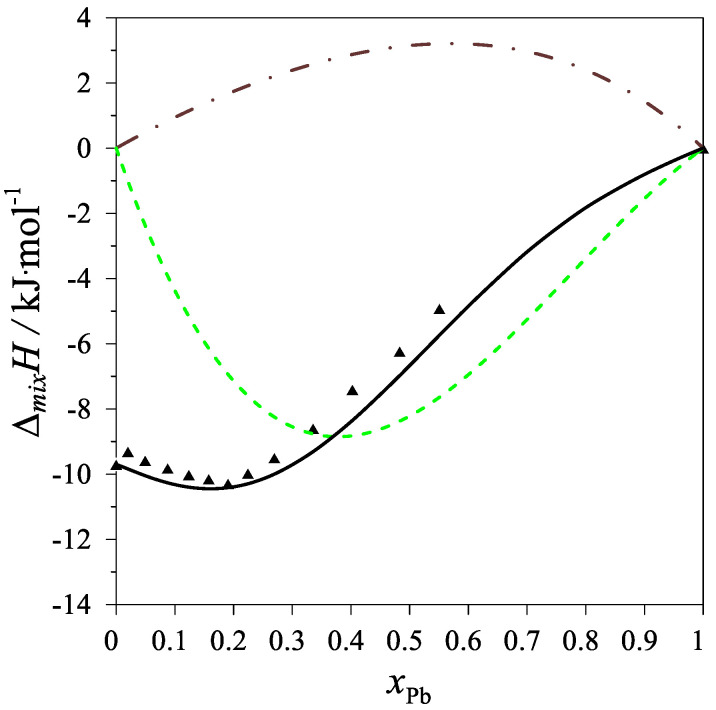
The integral mixing enthalpy of Ag–Mg–Pb liquid solutions for the intersection (Ag_0.25_Mg_0.75_)_1−x_Pb_x_ at 1116 K. Black line: data calculated with Equation (4), black triangles—experimental data. The dashed green line is the integral molar mixing enthalpy of Mg–Pb liquid alloys [12], and the brown dash-dotted line is the integral molar mixing enthalpy of Ag–Pb liquid alloys [17].

The determined values of the mixing enthalpy change for the Ag–Mg–Pb liquid solutions and those calculated with the use of Equation (4) and the parameters in Table 4 are presented in Figure 2 and Figure 3, where solid lines are the integral mixing enthalpy data calculated based on Equation (4) and the symbols show the experimental values obtained in this study.

By applying the parameters from Table 4 and Equation (4), the integral and partial mixing enthalpies for the Ag–Mg–Pb liquid solutions were calculated and are presented in Table 5.

**Figure 4:**
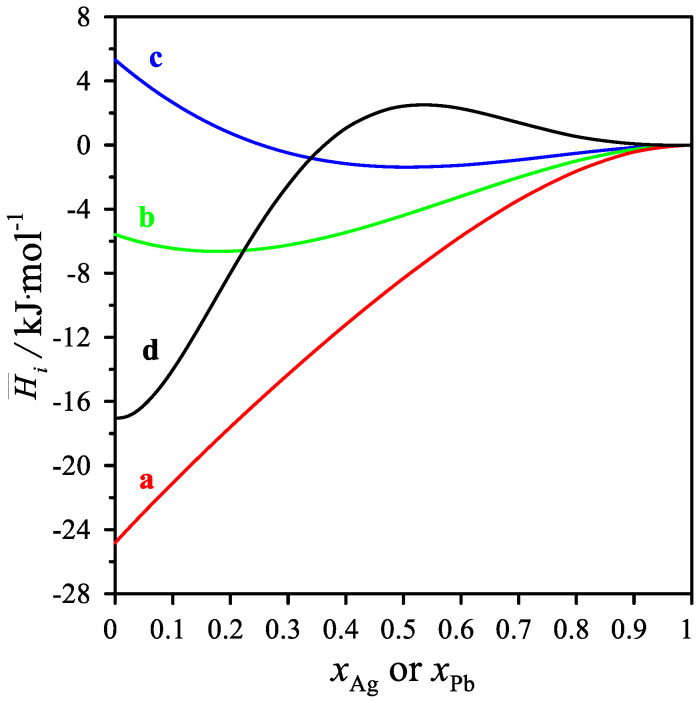
The partial molar mixing enthalpy Δ$\bar{H}$_i_ for the measured intersections. Δ$\bar{H}$ _Ag_ for (a) *x*_Mg_/*x*_Pb_ = 1/3, (b) *x*_Mg_/*x*_Pb_ = 1, (c) *x*_Mg_/*x*_Pb_ = 3 and Δ$\bar{H}$ _Pb_ for the measured intersections (d) *x*_Ag_/*x*_Mg_ = 1/3 at 1116 K.

As shown in Table 5 and in Figure 4, the partial mixing enthalpies of Ag and Pb have both positive and negative values. For the cross-sections *x*_Mg_/*x*_Pb_ = 1/3 and 1, the values of the partial silver mixing enthalpy decrease, reaching a minimum of −1.379 kJ/mol for the mole fraction *x*_Ag_ = 0.5 and −6.633 kJ/mol for the mole fraction *x*_Ag_ = 0.1916. On the other hand, the values of the partial mixing enthalpy of silver for the cross-section *x*_Mg_/x_Pb_ = 3 increase with increasing silver content in the alloy. For the cross-section *x*_Mg_/*x*_Ag_ = 3, the values of the partial mixing enthalpy of lead increase, reaching a maximum of 2.492 J/mol for the mole fraction *x*_Pb_ = 0.5506. The values of the partial mixing enthalpy of Mg in the measured cross-sections resulted in only negative values.

**Figure 5:**
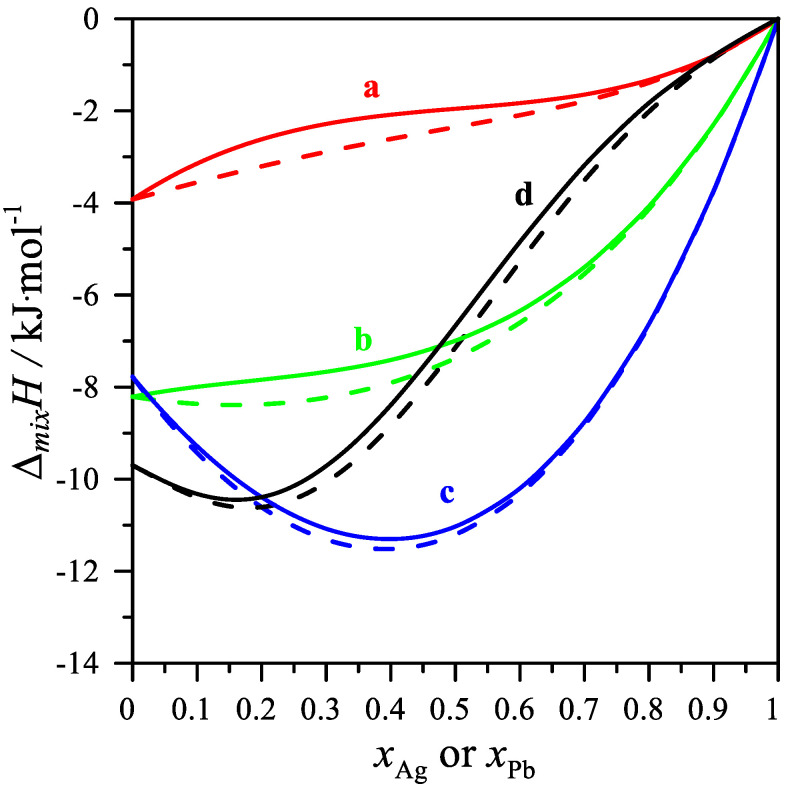
Comparison of the mixing enthalpy change calculated using the Muggianu model [46] (only binary systems; dashed lines) and using the same model with the ternary interaction parameter L_ijk_ shown in Table 4 (Equation (4); continuous lines); (a) *x*_Mg_/*x*_Pb_ = 1/3, (b) *x*_Mg_/*x*_Pb_ = 1, (c) *x*_Mg_/*x*_Pb_ = 3, (d) *x*_Ag_/*x*_Mg_ = 1/3.

The comparison of the data calculated by the Muggianu model is presented in Figure 5, and the calculation was performed by using only the thermodynamic properties of binary alloys with the thermodynamic properties calculated by using the ternary interaction parameters shown in Table 4.

For the starting alloys in the Mg–Pb system, the differences between the two variants are less than 0.7 kJ/mol. In the case of starting alloys of the Ag–Mg system, the differences between the two variants are less than 0.5 kJ/mol. The differences between the experimental data and those calculated by the Muggianu model with the ternary interaction parameters in the case of the studied liquid solutions are less than 1.5 kJ/mol.

## 4. Conclusions

This paper presents calorimetric measurements for the partial and integral molar mixing enthalpies of Ag–Mg–Pb liquid alloys. The determined results of the integral molar mixing enthalpies of liquid Ag–Mg–Pb alloys show negative deviations from the ideal solutions over the entire range of concentrations.

Based on our own calorimetric data for binary systems and the values of mixing enthalpy for the liquid Ag–Mg–Pb alloys measured in this study, the thermodynamic description of the ternary system was proposed in the form of the Muggianu model with the ternary interaction parameters that were calculated by the least square method. The counted standard deviation is equal to 0.582 kJ/mol.

Depending on the selected cross-section, the partial mixing enthalpies of Ag and Pb have both positive and negative values. On the other hand, the partial mixing enthalpy of Mg in the measured sections have only negative values.

The values of the mixing enthalpy calculated using the Muggianu model with the elaborated ternary parameters are in good agreement with the values obtained experimentally.

The observed differences between the values calculated by applying the Muggianu model with and without ternary interaction parameters are lower than 1 kJ/mol.

These are the first conducted experimental studies of the Ag–Mg–Pb liquid alloys and could be used in the future to optimize thermodynamic properties and phase diagram calculations.

## Figures and Tables

**Table 1:** Specifications of the applied materials.

Chemical Name	Source	Purity [Mass%]	Purification Method	Analysis Method
Magnesium	Alfa Aesar	99.9	None	Certified purity
Lead	POCH	99.999	None	Certified purity
Silver	Innovator Sp. z o.o.	99.9	None	Certified purity
Argon	Air Products	99.9999	None	Certified purity

**Table 2:** The integral mixing enthalpy of (Mg_0.25_Pb_0.75_)_1−x_Ag_x_, (Mg_0.50_Pb_0.50_)_1−x_Ag_x_, and (Mg_0.75_Pb_0.25_)_1−x_Ag_x_. Standard states: pure liquid metals.

Number of Dropped Moles of Ag [mol]	Heat Effect Δ*H*Signal·*K* [kJ]	Drop Enthalpy $H_{\mathrm{DISS}-\mathrm{Ag}}$ [kJ]	Mole Fraction *x*Ag	Integral Molar Enthalpy Δmix*H* [kJ/mol]	Partial Molar Enthalpy $\Delta {\bar{H}}_{\mathrm{Ag}}$ [kJ/mol]	Standard Uncertainties u(Δmix*H*) [kJ/mol]
$\mathrm{Series}\;A\;{\left({\mathrm{Mg}}_{0.25}{\mathrm{Pb}}_{0.75}\right)}_{1-x}{\mathrm{Ag}}_{x}:\;\mathrm{Atmosphere}:\;\mathrm{Argon}\;\mathrm{at}\;\mathrm{pressure}\;p=0.1\;\mathrm{MPa}.\;\mathrm{starting}\;\mathrm{amount}:\;n_{\mathrm{Mg}}=0.003197\;\mathrm{mol};\;n_{\mathrm{Pb}}=0.009590\;\mathrm{mol};\;K=0.000003815\;\mathrm{kJ}/µ\mathrm{Vs};\;T_{D}=298\;K;\;T_{M}=1116\;K;\;\mathrm{kJ}/\mathrm{mol};\;\Delta H_{\mathrm{Ag}}^{T_{D}\to T_{M}}$$=33.7485\;\mathrm{kJ}/\mathrm{mol};\;\Delta H_{\mathrm{Mg}}^{T_{D}\to T_{M}}$$=32.8525\;\mathrm{kJ}/\mathrm{mol};\;\Delta H_{\mathrm{Pb}}^{T_{D}\to T_{M}}$=28.6608 kJ/mol; $\Delta_{\mathrm{mix}}H_{{\mathrm{Mg}}_{0.25}{\mathrm{Pb}}_{0.75}}$$=-4.2\;\mathrm{kJ}/\mathrm{mol}.\;\mathrm{Standard}\;\mathrm{uncertainties}:\;u(n_{\mathrm{Ag}})=0.\;0.0000009\;\mathrm{mol};\;u(n_{\mathrm{Mg}})=0.000004\;\mathrm{mol};\;u(n_{\mathrm{Pb}})=0.0000005;\;u(T_{D})=1\;K;\;u(T_{M})=1\;K;\;u(p)=10\;\mathrm{kPa};\;u(K)=0.000000035\;\mathrm{kJ}/\mu \mathrm{Vs};\;u($$\Delta_{\mathrm{mix}}H_{{\mathrm{Mg}}_{0.25}{\mathrm{Pb}}_{0.75}})$ = 0.04 kJ/mol.						
0.000929	0.034250	0.003	0.0677	−3.7	3.1	0.06
0.000959	0.033922	0.002	0.1287	−3.4	1.6	0.08
0.000996	0.035925	0.002	0.1840	−3.0	2.3	0.11
0.001044	0.036470	0.001	0.2350	−2.7	1.2	0.13
0.001137	0.039690	0.001	0.2837	−2.5	1.1	0.15
0.001119	0.038816	0.001	0.3260	−2.3	0.9	0.16
0.001072	0.039129	0.003	0.3620	−2.0	2.8	0.18
0.001115	0.039747	0.002	0.3956	−1.8	1.9	0.20
0.001077	0.038824	0.002	0.4249	−1.6	2.3	0.22
0.001353	0.049990	0.004	0.4579	−1.3	3.2	0.24
$\mathrm{Series}\;B\;{\left({\mathrm{Mg}}_{0.50}{\mathrm{Pb}}_{0.50}\right)}_{1-x}{\mathrm{Ag}}_{x}:\;\mathrm{Atmosphere}:\;\mathrm{Argon}\;\mathrm{at}\;\mathrm{pressure}\;p=0.1\;\mathrm{MPa}.\;\mathrm{starting}\;\mathrm{amount}:\;n_{\mathrm{Mg}}=0.009056\;\mathrm{mol};\;n_{\mathrm{Pb}}=0.009057\;\mathrm{mol};\;K=0.000003394\;\mathrm{kJ}/µ\mathrm{Vs};\;T_{D}=298\;K;\;T_{M}=1116\;K;\;\mathrm{kJ}/\mathrm{mol};\;\Delta H_{\mathrm{Ag}}^{T_{D}\to T_{M}}$$=33.7485\;\mathrm{kJ}/\mathrm{mol};\;\Delta H_{\mathrm{Mg}}^{T_{D}\to T_{M}}$$=32.8525\;\mathrm{kJ}/\mathrm{mol};\;\Delta H_{\mathrm{Pb}}^{T_{D}\to T_{M}}$=28.6608 kJ/mol; $\Delta_{\mathrm{mix}}H_{{\mathrm{Mg}}_{0.50}{\mathrm{Pb}}_{0.50}}$$=-7.7\;\mathrm{kJ}/\mathrm{mol}.\;\mathrm{Standard}\;\mathrm{uncertainties}:\;u(n_{\mathrm{Ag}})=0.\;0.0000009\;\mathrm{mol};\;u(n_{\mathrm{Mg}})=0.000004\;\mathrm{mol};\;u(n_{\mathrm{Pb}})=0.0000005;\;u(T_{D})=1\;K;\;u(T_{M})=1\;K;\;u(p)=10\;\mathrm{kPa};\;u(K)=0.000000060\;\mathrm{kJ}/\mu \mathrm{Vs};\;u($$\Delta_{\mathrm{mix}}H_{{\mathrm{Mg}}_{0.50}{\mathrm{Pb}}_{0.50}})$ = 0.16 kJ/mol.						
0.001148	0.032040	−0.007	0.0596	−7.6	−5.8	0.19
0.000980	0.023487	−0.010	0.1051	−7.7	−9.8	0.21
0.000974	0.024040	−0.009	0.1462	−7.8	−9.1	0.23
0.001192	0.028836	−0.011	0.1916	−7.8	−9.6	0.25
0.001446	0.035855	−0.013	0.2407	−7.9	−9.0	0.28
0.001457	0.040151	−0.009	0.2844	−7.8	−6.2	0.30
0.001494	0.044598	−0.006	0.3243	−7.6	−3.9	0.33
0.001521	0.040084	−0.011	0.3606	−7.6	−7.4	0.36
0.001724	0.046074	−0.012	0.3973	−7.6	−7.0	0.39
0.001727	0.049376	−0.009	0.4300	−7.4	−5.2	0.41
0.001647	0.050232	−0.005	0.4581	−7.2	−3.3	0.44
0.001765	0.051050	−0.009	0.4853	−7.1	−4.8	0.47
$\mathrm{Series}\;C\;{\left({\mathrm{Mg}}_{0.75}{\mathrm{Pb}}_{0.25}\right)}_{1-x}{\mathrm{Ag}}_{x}:\;\mathrm{Atmosphere}:\;\mathrm{Argon}\;\mathrm{at}\;\mathrm{pressure}\;p=0.1\;\mathrm{MPa}.\;\mathrm{starting}\;\mathrm{amount}:\;n_{\mathrm{Mg}}=0.025624\;\mathrm{mol};\;n_{\mathrm{Pb}}=0.008543\;\mathrm{mol};\;K=0.000003325\;\mathrm{kJ}/µ\mathrm{Vs};\;T_{D}=298\;K;\;T_{M}=1116\;K;\;\mathrm{kJ}/\mathrm{mol};\;\Delta H_{\mathrm{Ag}}^{T_{D}\to T_{M}}$$=33.7485\;\mathrm{kJ}/\mathrm{mol};\;\Delta H_{\mathrm{Mg}}^{T_{D}\to T_{M}}$$=32.8525\;\mathrm{kJ}/\mathrm{mol};\;\Delta H_{\mathrm{Pb}}^{T_{D}\to T_{M}}$=28.6608 kJ/mol; $\Delta_{\mathrm{mix}}H_{{\mathrm{Mg}}_{0.75}{\mathrm{Pb}}_{0.25}}$$=-7.5\;\mathrm{kJ}/\mathrm{mol}.\;\mathrm{Standard}\;\mathrm{uncertainties}:\;u(n_{\mathrm{Ag}})=0.\;0.0000009\;\mathrm{mol};\;u(n_{\mathrm{Mg}})=0.000004\;\mathrm{mol};\;u(n_{\mathrm{Pb}})=0.0000005;\;u(T_{D})=1\;K;\;u(T_{M})=1\;K;\;u(p)=10\;\mathrm{kPa};\;u(K)=0.000000062\;\mathrm{kJ}/\mu \mathrm{Vs};\;u($$\Delta_{\mathrm{mix}}H_{{\mathrm{Mg}}_{0.75}{\mathrm{Pb}}_{0.25}})$ = 0.44 kJ/mol.						
0.0011199	0.003841	−0.034	0.0317	−8.2	−30.3	0.44
0.0014406	0.007875	−0.041	0.0697	−9.0	−28.3	0.44
0.0016789	0.010824	−0.046	0.1104	−9.8	−27.3	0.45
0.0020599	0.018174	−0.051	0.1557	−10.6	−24.9	0.46
0.0020673	0.023159	−0.047	0.1967	−11.2	−22.5	0.47
0.0021758	0.032061	−0.041	0.2358	−11.5	−19.0	0.48
0.0022833	0.034419	−0.043	0.2729	−11.9	−18.7	0.49
0.0022861	0.034784	−0.042	0.3067	−12.2	−18.5	0.51
0.0029063	0.049882	−0.048	0.3453	−12.4	−16.6	0.52
0.0031056	0.057853	−0.047	0.3820	−12.6	−15.1	0.54
0.0032734	0.065761	−0.045	0.4166	−12.7	−13.7	0.56
0.0037230	0.085006	−0.041	0.4515	−12.6	−10.9	0.59

**Table 3:** The integral mixing enthalpy of (Ag_0.25_Mg_0.75_)_1−x_Pb_x_. Standard states: pure liquid metals.

Number of Dropped Moles of Pb [mol]	Heat Effect Δ*H*Signal·*K* [kJ]	Drop Enthalpy $H_{\mathrm{DISS}-\mathrm{Pb}}$ [kJ]	Mole Fraction *x*Pb	Integral Molar Enthalpy Δmix*H* [kJ/mol]	Partial Molar Enthalpy $\Delta {\bar{H}}_{\mathrm{Pb}}$ [kJ/mol]	Standard Uncertainties u(Δmix*H*) [kJ/mol]
$\mathrm{Series}\;D\;{\left({\mathrm{Ag}}_{0.25}{\mathrm{Mg}}_{0.75}\right)}_{1-x}{\mathrm{Pb}}_{x}:\;\mathrm{Atmosphere}:\;\mathrm{Argon}\;\mathrm{at}\;\mathrm{pressure}\;p=0.1\;\mathrm{MPa}.\;\mathrm{starting}\;\mathrm{amount}:\;n_{\mathrm{Ag}}=0.004222\;\mathrm{mol};\;n_{\mathrm{Mg}}=0.024332\;\mathrm{mol};\;K=0.000003480\;\mathrm{kJ}/µ\mathrm{Vs};\;T_{D}=298\;K;\;T_{M}=1116\;K;\;\mathrm{kJ}/\mathrm{mol};\;\Delta H_{\mathrm{Ag}(s)}^{T_{D}\to T_{M}}$$=22.7234\;\mathrm{kJ}/\mathrm{mol};\;\Delta H_{\mathrm{Ag}(l)}^{T_{D}\to T_{M}}$$=33.7485\;\mathrm{kJ}/\mathrm{mol};\;\Delta H_{\mathrm{Mg}}^{T_{D}\to T_{M}}$=32.8525 kJ/mol; $\Delta H_{\mathrm{Pb}}^{T_{D}\to T_{M}}$=28.6608 kJ/mol; $\Delta_{\mathrm{mix}}H_{{\mathrm{Ag}}_{0.25}{\mathrm{Mg}}_{0.75}}$$=-9.7\;\mathrm{kJ}/\mathrm{mol}.\;\mathrm{Standard}\;\mathrm{uncertainties}:\;u(n_{\mathrm{Ag}})=0.\;0.0000009\;\mathrm{mol};\;u(n_{\mathrm{Mg}})=0.000004\;\mathrm{mol};\;u(n_{\mathrm{Pb}})=0.0000005;\;u(T_{D})=1\;K;\;u(T_{M})=1\;K;\;u(p)=10\;\mathrm{kPa};\;u(K)=0.000000067\;\mathrm{kJ}/\mu \mathrm{Vs};\;u($$\Delta_{\mathrm{mix}}H_{{\mathrm{Ag}}_{0.25}{\mathrm{Mg}}_{0.75}})$ = 0.54 kJ/mol.						
0.0006636	−0.012041	−0.031	0.0200	−9.3	−46.8	0.55
0.0010212	0.010405	−0.019	0.0494	−9.6	−18.5	0.56
0.0014334	0.018966	−0.022	0.0877	−9.8	−15.4	0.57
0.0014633	0.020177	−0.022	0.1237	−10.0	−14.9	0.58
0.0014899	0.022766	−0.020	0.1576	−10.1	−13.4	0.59
0.0015642	0.023316	−0.022	0.1905	−10.3	−13.8	0.60
0.0017505	0.045275	−0.005	0.2244	−10.0	−2.8	0.62
0.0025801	0.069316	−0.005	0.2695	−9.5	−1.8	0.65
0.0044069	0.128546	0.002	0.3354	−8.6	0.5	0.70
0.0054725	0.174444	0.018	0.4024	−7.4	3.2	0.76
0.0084517	0.253278	0.011	0.4829	−6.2	1.3	0.84
0.0094570	0.306745	0.036	0.5506	−4.9	3.8	0.92

**Table 4:** The binary Lki,jLiquid and ternary interaction parameters Lk123Liquid in Equation (4) for the Ag–Mg–Pb liquid alloys.

System	Binary Lki,jLiquid and Ternary Lk123Liquid Parameters [J/mol]	References
Ag–Mg	L0Ag,MgLiquid=−53,346.5 L1Ag,MgLiquid=−3694 L2Ag,MgLiquid=−905.8	[10]
Ag–Pb	L0Ag,PbLiquid=12,902.274	[17]
	L1Ag,PbLiquid=−4008.088 L2Ag,PbLiquid=−2576.139	
Mg–Pb	L0Mg,PbLiquid=−39,272.641-5.773712·*T*	[12]
	L1MgPbLiquid=−43,546.211-16.706741·*T*	
	L2MgPbLiquid=−4329.298-8.616737·*T*	
	L3MgPbLiquid=17,406.133	
	L4MgPbLiquid=4967.802	
Ag–Mg–Pb	L1Ag, Mg,PbLiquid=4700.8075	This study
	L2Ag, Mg,PbLiquid=518.60761	
	L3Ag, Mg,PbLiquid=39,007.392	

**Table 5:** The partial and integral functions of Ag–Mg–Pb liquid alloys.

*x*_Ag_	*x*_Mg_	*x*_Pb_	$\Delta {\bar{H}}_{\mathrm{Ag}}$	$\Delta {\bar{H}}_{\mathrm{Mg}}$	$\Delta {\bar{H}}_{\mathrm{Pb}}$	Δ_mix_*H*
			kJ/mol			
(Mg_0.25_Pb_0.75_)_1−xAgx_ alloys at *T* = 1116 K						
0	0.25	0.75	5.318	−17.286	0.532	−3.923
0.05	0.2375	0.7125	3.880	−18.909	1.121	−3.498
0.1	0.225	0.675	2.646	−20.519	1.790	−3.144
0.2	0.2	0.6	0.744	−23.735	3.302	−2.617
0.3	0.175	0.525	−0.490	−27.000	4.930	−2.284
0.4	0.15	0.45	−1.161	−30.386	6.529	−2.084
0.5	0.125	0.375	−1.379	−33.969	7.949	−1.955
0.6	0.1	0.3	−1.261	−37.832	9.027	−1.832
0.7	0.075	0.225	−0.929	−42.063	9.594	−1.647
0.8	0.05	0.15	−0.514	−46.756	9.465	−1.330
0.9	0.025	0.075	−0.155	−52.013	8.444	−0.806
1	0	0	0	−57.946	6.318	0
(Mg_0.50_Pb_0.50_)_1−x_Ag_x_ alloys at *T* = 1116 K						
0	0.5	0.5	−5.584	−14.433	−1.982	−8.207
0.05	0.475	0.475	−6.112	−15.669	−0.720	−8.090
0.1	0.45	0.45	−6.450	−16.889	0.553	−7.996
0.2	0.4	0.4	−6.624	−19.354	3.066	−7.840
0.3	0.35	0.35	−6.246	−21.982	5.427	−7.669
0.4	0.3	0.3	−5.455	−24.926	7.500	−7.410
0.5	0.25	0.25	−4.392	−28.340	9.152	−6.993
0.6	0.2	0.2	−3.199	−32.379	10.248	−6.346
0.7	0.15	0.15	−2.018	−37.201	10.651	−5.396
0.8	0.1	0.1	−0.995	−42.964	10.223	−4.070
0.9	0.05	0.05	−0.273	−49.826	8.825	−2.296
1	0	0	0	−57.946	6.318	0
(Mg_0.75_Pb_0.25_)_1−x_Ag_x_ alloys at *T* = 1116 K						
0	0.75	0.25	−24.819	−2.974	−22.182	−7.776
0.05	0.7125	0.2375	−22.916	−4.358	−18.227	−8.579
0.1	0.675	0.225	−21.085	−5.781	−14.551	−9.285
0.2	0.6	0.2	−17.600	−8.792	−7.987	−10.393
0.3	0.525	0.175	−14.311	−12.114	−2.420	−11.077
0.4	0.45	0.15	−11.210	−15.881	2.177	−11.304
0.5	0.375	0.125	−8.320	−20.248	5.791	−11.030
0.6	0.3	0.1	−5.699	−25.395	8.372	−10.201
0.7	0.225	0.075	−3.433	−31.518	9.838	−8.757
0.8	0.15	0.05	−1.634	−38.831	10.080	−6.627
0.9	0.075	0.025	−0.437	−47.560	8.960	−3.736
1	0	0	0	−57.946	6.318	0
(Ag_0.25_Mg_0.75_)_1−x_Pb_x_ alloys at *T* = 1116 K						
0.25	0.75	0	−29.880	−2.972	−17.047	−9.699
0.2375	0.7125	0.05	−27.155	−3.919	−16.231	−10.053
0.225	0.675	0.1	−23.995	−5.219	−14.018	−10.323
0.2	0.6	0.2	−17.600	−8.792	−7.987	−10.393
0.175	0.525	0.3	−12.104	−13.056	−2.478	−9.716
0.15	0.45	0.4	−7.707	−17.004	1.042	−8.391
0.125	0.375	0.5	−3.991	−19.697	2.432	−6.669
0.1	0.3	0.6	−0.384	−20.577	2.273	−4.847
0.075	0.225	0.7	3.502	−19.653	1.398	−3.181
0.05	0.15	0.8	7.656	−17.564	0.532	−1.826
0.025	0.075	0.9	11.581	−15.514	0.075	−0.806
0	0	1	14.334	−15.079	0	0

## Data Availability

Raw data is available upon request.
